# Bacterial bioaugmentation for improving methane and hydrogen production from microalgae

**DOI:** 10.1186/1754-6834-6-92

**Published:** 2013-07-01

**Authors:** Fan Lü, Jiaqi Ji, Liming Shao, Pinjing He

**Affiliations:** 1 State Key Laboratory of Pollution Control and Resource Reuse, Tongji University, Shanghai 200092, China; 2 Institute of Waste Treatment and Reclamation, Tongji University, Shanghai 200092, China; 3 Centre for the Technology Research and Training on Household Waste in Small Towns & Rural Area, Ministry of Housing and Urban–rural Development of PR. China (MOHURD), Beijing, China

## Abstract

**Background:**

The recalcitrant cell walls of microalgae may limit their digestibility for bioenergy production. Considering that cellulose contributes to the cell wall recalcitrance of the microalgae *Chlorella vulgaris*, this study investigated bioaugmentation with a cellulolytic and hydrogenogenic bacterium, *Clostridium thermocellum*, at different inoculum ratios as a possible method to improve CH_4_ and H_2_ production of microalgae.

**Results:**

Methane production was found to increase by 17?~?24% with the addition of *C*. *thermocellum*, as a result of enhanced cell disruption and excess hydrogen production. Furthermore, addition of *C*. *thermocellum* enhanced the bacterial diversity and quantities, leading to higher fermentation efficiency. A two-step process of addition of *C*. *thermocellum* first and methanogenic sludge subsequently could recover both hydrogen and methane, with a 9.4% increase in bioenergy yield, when compared with the one-step process of simultaneous addition of *C*. *thermocellum* and methanogenic sludge. The fluorescence peaks of excitation-emission matrix spectra associated with chlorophyll can serve as biomarkers for algal cell degradation.

**Conclusions:**

Bioaugmentation with *C*. *thermocellum* improved the degradation of *C*. *vulgaris* biomass, producing higher levels of methane and hydrogen. The two-step process, with methanogenic inoculum added after the hydrogen production reached saturation, was found to be an energy-efficiency method for hydrogen and methane production.

## Background

Microalgae have enormous potential as a source for biofuel and bioenergy production due to their high photosynthetic efficiencies, high growth rates, and characteristics of not requiring external organic carbon supply. Anaerobic digestion of algal biomass to biogas containing methane or hydrogen is one of the most energy-efficient and environmentally beneficial technologies [1]. The process is highly dependent on both substrate degradability as well as environmental conditions which regulate the microbial activity [2].

Anaerobic digestion could be carried out on microalgal residues after lipid extraction [3-6] or directly on freshly collected algae. With regard to the latter, the resistance of the microalgal cell wall could be one of the limiting factors for cell digestibility [7,8]. The cell wall of some microalgal species such as *Chlorella* sp. and *Scenedesmus* sp. is known to contain recalcitrant cellulose [9], which could protect the microalgae against enzyme attack, thus restricting algal biodegradability [3,10]. Lakaniemi et al. [11] found that only approximately 50% of *Chlorella vulgaris* biomass was degraded during methanogenic fermentation. Various mechanical (high-pressure homogenization, bead beating), physical (ultrasonication), thermal, and chemical (acids, bases, and oxidizing agents) pretreatment methods have been investigated to improve the digestion efficiency [3,8,12-14]. However, although these pretreatment technologies could enhance methane production from algae with thick cell wall, the energy cost of pretreatment is high. For example, the amount of energy consumed in heating and pretreatment was found to be higher than or equal to the corresponding energy gain from increased methane production [3,15,16]. Besides, the use of thermochemical pretreatment may also lead to a possible formation of inhibitory substances (e.g. furfurals) [17]. Enzymatic hydrolysis is a well-known biological pretreatment process. Sander and Murthy [18] found that cell walls of mixed algae are susceptible to degradation by cellulase and lipase. Ehimen et al. [13] reported a pretreatment process of addition of a combined enzyme mixture and individual enzymes to the *Rhizoclonium* biomass prior to anaerobic digestion. The researchers observed that the enzymatic pretreatment led to greater methane conversions than the mechanical methods, and that the action of cellulase resulted in maximum methane yield, when compared with that of other enzymes. However, enzymes are usually only effective at the initial stage after addition and become inactive soon afterwards. Comparatively, living bacteria can continuously hydrolyze the materials through growth and proliferation. Nevertheless, appropriate bacterial species should be carefully selected to be effective for microalgae hydrolysis and be compatible with subsequent or synchronous anaerobic digestion.

Considering that cellulose contributes to the cell wall recalcitrance in the microalgae *C*. *vulgaris*, this study investigated bioaugmentation with a thermophilic, anaerobic, cellulolytic, and hydrogenogenic bacterium, *Clostridium thermocellum*, which is also available from cellulose-fed anaerobic digester [19], as a possible method to improve the degradation of *C*. *vulgaris* biomass to enhance the efficiency of methane and hydrogen production. To our best knowledge, the present study is the first report on improving *C*. *vulgaris* degradation by bioaugmentation using *C*. *thermocellum*.

## Results

### Methane and hydrogen production

**Figure 1:**
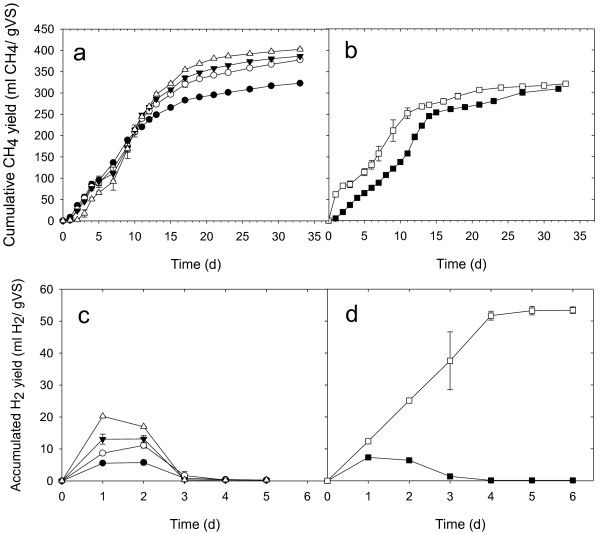
**Methane and hydrogen production from *C. vulgaris *biomass. (a)** cumulative methane yield in Series 1; **(b)** cumulative methane yield in Series 2; **(c)** accumulated hydrogen yield in Series 1; **(d)** accumulated hydrogen yield in Series 2, where inoculum ratios of *C. thermocellum* were 0% (●), 1%(○), 5%(▼), 10%(△) and (■) for one-step experiment, (□) for two-step experiment. Error bar represents the data range of duplicate test.

As shown in Figure 1a, all of the observed cumulative methane production increased steadily after a short lag phase, and the plateau phase was reached at approximately 35 days. Gompertz modeling (Table 1) revealed that the highest methane yield from 2 g VS/L of *C*. *vulgaris* without *C*. *thermocellum* in Series 1 was 318 ml/g VS. There was a clear difference in methane production after addition of *C*. *thermocellum*. The highest methane yields for the inoculum ratios of 1%, 5%, and 10% of *C*. *thermocellum* were 376, 388, and 403 ml/g VS, respectively. Correspondingly, the maximum methane production rate was found to increase from 23.11 to 33.14 ml/g VS/day, and the lag time was noted to increase from 0.83 to 3.61 days when the inoculum ratio of *C*. *thermocellum* was increased from 0% to 10%. However, when the concentration of algal biomass was increased to 3 g VS/L in Series 2, the highest methane yield and maximum methane production rate were much lower than those noted for the corresponding treatment in Series 1 with the same inoculum ratio of 5% of *C*. *thermocellum* (Figure 1b). Nevertheless, when compared with the one-step treatment, in the two-step treatment of Series 2, the maximum methane production rate was increased by 9% and the lag time was decreased by 2.37 days.

**Table 1:** Calculated result using the modified gompertz equation for the cumulative methane production

**Series**	**Treatment**^**a**^	**Ultimate methane yield*P*(ml/g-VS)**	**Maximum methane production rate*R***_***max***_**(ml/g VS?·?d)**	**Lag phase** ***λ*(d)**	**R**^**2**^
Series 1	0%	317.8?±?3.0	23.11?±?0.84	0.83?±?0.20	0.996
	1%	375.4?±?4.4	25.36?±?1.08	1.62?±?0.25	0.996
	5%	387.7?±?5.1	27.38?±?1.32	2.07?±?0.28	0.994
	10%	402.8?±?3.8	33.14?±?1.30	3.61?±?0.21	0.997
Series 2	One-step	316.6?±?5.3	20.36?±?1.21	2.37?±?0.36	0.990
	Two-step	320.6?±?6.5	22.38?±?1.78	0?±?0.44	0.986

a: In series 1, *Clostridium thermocellum* was added by 0%, 1%, 5% and 10% (v/v) with 2 g VS/L of algal biomass and 3 g VS/L of methanogenic sludge. In series 2, 5% *C. thermocellum* was added with 3 g VS/L of algal biomass and 2 g VS/L of methanogenic sludge for one-step. For two-step, 3 g VS/L of algal biomass was first incubated with 5% (v/v) of *C. thermocellum* for hydrogen production and 2 g VS/L of methanogenic sludge was subsequently added to produce methane. The parameter standard error was obtained from the weighted regression.

Hydrogen is a key intermediate during anaerobic digestion as well as a product synthesized by *C*. *thermocellum*. Hence, in the enrichment cultures of Series 1 with mixed inoculum and in the one-step treatment of Series 2, hydrogen was accumulated in the first few days and then was rapidly consumed by methanogens (Figure 1c). Hydrogen production increased with the increase in the inoculum ratio of *C*. *thermocellum*. Comparatively, in the two-step treatment of Series 2, the hydrogen produced in the first step with only *C*. *thermocellum* was further consumed and reached a maximum plateau value of 53.4 ml H_2_/g VS after 5 days (Figure 1d), equivalent to 0.167 mol/mol of the corresponding methane production in the second step.

### Production of ethanol and volatile fatty acids (VFAs)

**Figure 2:**
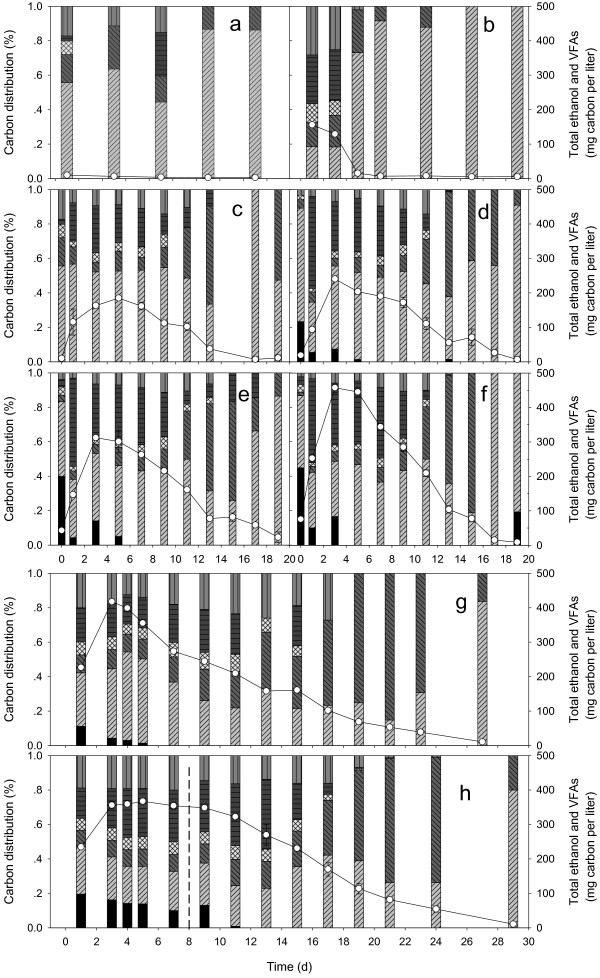
**Ethanol and VFAs production. (a)** The control with only granular sludge and without microalgae; **(b)** the control with granular sludge and 5% (v/v) of *C. thermocellum*-containing culture medium; **(c)** 0%, **(d)** 1%, **(e)** 5% and **(f)** 10% (v/v) of *C. thermocellum* in Series 1; **(g)(h)** one-step and two-step experiments in series2. ethanol, acetate, propionate, isobutyrate, butyrate, isovalerate and (○) total amount of ethanol and VFAs. The dashed in **(h)** showed the beginning of the second step.

Ethanol and VFAs were noted to be the main products in the acidogenic hydrogen-producing fermentation. The addition of *C*. *thermocellum* resulted in rapid production of ethanol from the microalgae in the initial 3 days (Figure 2). In Series 1, the ethanol accumulated to a maximum concentration, increasing from 0 to 77 mg carbon per liter, with the increasing *C*. *thermocellum* inoculum ratio from 0% to 10%, and then was rapidly consumed (Figure 2c-f). In the two-step treatment of Series 2 with higher microalgae concentration, the ethanol concentration remained at 51–60 mg carbon per liter in the first step until granular sludge was added (Figure 2h). The levels of total VFAs also increased from 185 to 457 mg carbon per liter with the increase in *C*. *thermocellum* inoculum ratio from 0% to 10%. In all the treatments, acetate, propionate, and butyrate were the dominate metabolites. Acetate and butyrate accounted for more than 70% of the total VFAs before a shift towards acetate and propionate pathways after 10 days. In Series 2, treatments with higher proportion of algal biomass showed relatively high levels of VFAs (418 and 367 mg carbon per liter), in which a significant level of isovalerate was detected (Figure 2g, h).

### Microalgal degradation and bacterial growth monitored by fluorescent method

**Figure 3:**
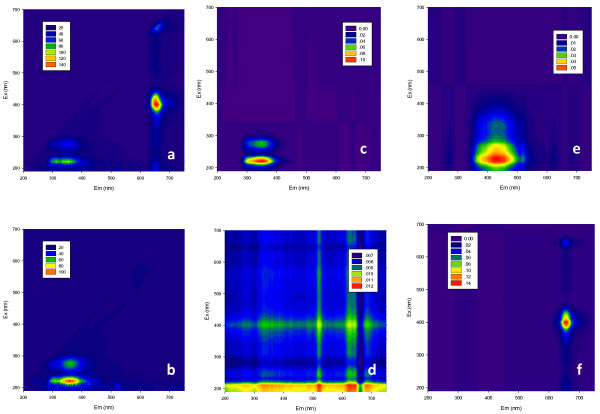
**Excitation-emission matrix (EEM) profiles and four components extracted from parallel factor analysis (PARAFAC). (a)** EEM profile of *C. vulgaris* microalgae; **(b)** EEM profile of *C. thermocellum*; **(c)** Component 1; **(d)** Component 2; **(e)** Component 3; **(f)** Component 4.

Algal cells contain specific fluorescent biochemical components (e.g. chlorophyll), and therefore exhibit different excitation-emission matrix (EEM) profiles [20]. In the present study, *C*. *vulgaris* was found to demonstrate specific EEM profiles (Figure 3a) with five distinct fluorescent peaks (Ex/EM 400/660, 400/682, 400/625, 420/652, and 650/662), which might be attributed to chlorophyll *a* and *b*[20,21]. On the other hand, the EEM profile of *C*. *thermocellum* culture medium was completely different with two distinct peaks (Ex/EM 220/354, 270/354) (Figure 3b). Hence, the EEM profiles along the digestion process were collected to monitor the degradation of *C*. *vulgaris*, and were analyzed by parallel factor analysis (PARAFAC) method to resolve the EEM signals of the unknown samples from those of any overlapping and uncalibrated interferents. The core consistencies were 100%, 100%, 98.9%, 98.2%, 74.5%, 64.5%, and 7% for component numbers ranging from 1 to 7, respectively. Therefore, the optimal number of components was set to 4 for this model. The EEM contours of Components 1–4 are shown in Figure 3c–f, respectively. On comparing the basic EEM profiles of *C*. *vulgaris* and *C*. *thermocellum* culture medium, the following could be observed: Component 4 with the highest fluorescence was assigned to the distinct fluorophores from *C*. *vulgaris*; Component 1 was primarily contributed by *C*. *thermocellum* and partly by *C*. *vulgaris*; Component 3 was assigned to the soluble microbial products (SMP) generated from anaerobic digestion of the inoculated granular sludge; and Component 2 with low fluorescence intensity was the systematic background of buffer solutions and instruments.

**Figure 4:**
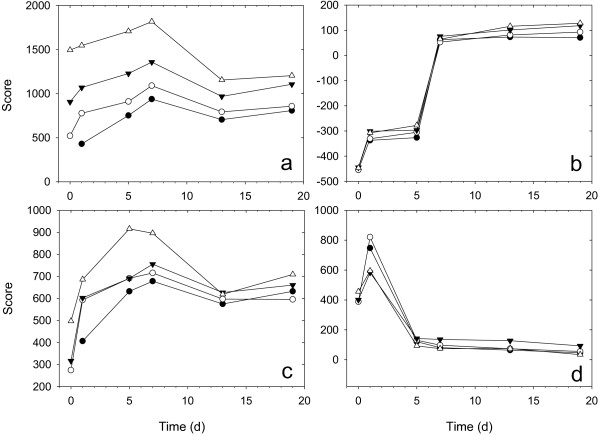
**Scores of PARAFAC components in different samples from series 1. (a)** Component 1; **(b)** Component 2; **(c)** Component 3; **(d)** Component 4, where inoculum ratios of *C. thermocellum* were 0% (●), 1%(○), 5%(▼), 10%(△).

The scores of each PARAFAC component in different samples of Series 1 are presented in Figure 4. It can be noted that the scores of Component 1 representing *C*. *thermocellum* significantly increased with the *C*. *thermocellum* inoculum ratio, suggesting that the bacteria kept growing on the substrate of *C*. *vulgaris* during the initial 7 days. Component 3 representing SMP also had higher scores at higher *C*. *thermocellum* inoculum ratio, implying that the growth of methanogenic inoculum was promoted by the addition of *C*. *thermocellum*. The scores of Component 4 representing microalgal fluorophores increased quickly on the first day and then decreased to a low value from Day 5 onwards, indicating rapid breakage and hydrolysis of the microalgal cell wall in 24 h, resulting in the release of intracellular fluorophores. The released fluorophores were instantly fermented into low-molecular intermediates in 5 days, and the fermentation rate was higher in treatments with 5% and 10% *C*. *thermocellum*.

### Electron microscopic observation of microalgal cell degradation

**Figure 5:**
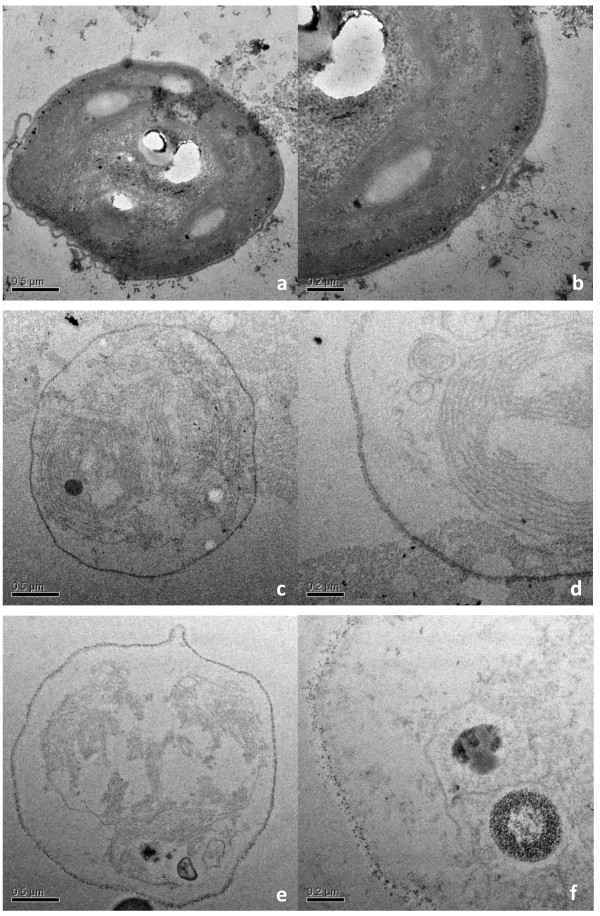
**Transmission electron micrographs of *C. vulgaris *during the cell degradation. (a**, **b)** Early stage; **(c**, **d)** medium stage; and **(e**, **f)** later stage. Scale bar 0.5 μm **(a**, **c**, **e)**, 0.2 μm **(b**, **d**, **f)**.

The *C*. *vulgaris* cells were observed under transmission electron microscopy (TEM). Samples taken from the treatment with 10% (v/v) *C*. *thermocellum* at 0, 7, and 15 days are shown in Figure 5. The cells were about 2 μm in diameter. The major portion of the cell was occupied by a C-shaped chloroplast made up of an array of photosynthetic lamellae. The initial cell was filled with cytoplasm and remained enclosed by the cell wall (Figure 5a, b). With the degradation of the cell, the cell wall was slightly damaged, while the organelles were nearly intact (Figure 5c, d). Subsequently, the organelles and cell walls were all broken into pieces (Figure 5e, f). TEM studies revealed that the cell wall played a significant role in resisting the attack of microorganisms. Likewise, TEM observations of other treatments exhibited a similar trend of *C*. *vulgaris* degradation, but the degradation rates were slightly lower.

### Automated ribosomal intergenic spacer analysis (ARISA)

**Figure 6:**
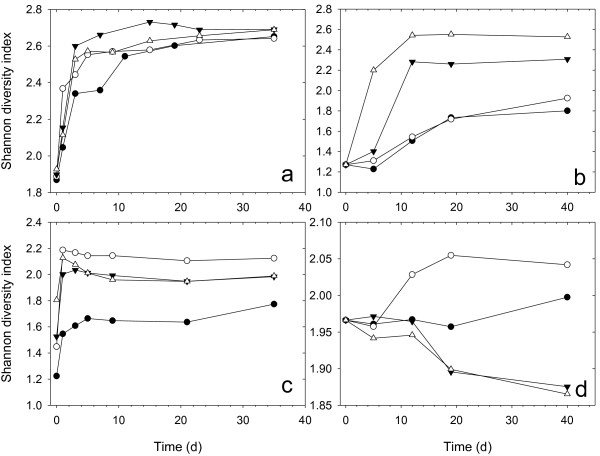
**Shannon diversity. (a)** Bacteria in liquid phase; **(b)** bacteria in solid phase; **(c)** methanogen in liquid phase; **(d)** methanogen in solid phase, where inoculum ratios of *C. thermocellum* were 0% (●), 1%(○), 5%(▼), 10%(△).

Shannon diversity index was used to analyze the ARISA profiles (Additional file 1: Figure S1-S4) to estimate the diversity of the microbial community (Figure 6) in the liquid and solid phase of the samples, respectively. For bacteria in liquid phase, the *H* values of the treatments with *C*. *thermocellum* were significantly higher than those of the treatment without *C*. *thermocellum* during the initial 7 days and the *H* value of the treatment with 5% inoculum ratio was the highest. In solid phase, the highest *H* values were found for treatment with 10% *C*. *thermocellum*, followed by those for treatment with 5% inoculum ratio. However, the *H* values of treatments with 1% and 0% *C*. *thermocellum* inoculum ratios were quite similar, and much lower than those of treatments with 10% and 5% inoculum ratios.

Similarly, for methanogens in liquid phase, the *H* values of treatments with *C*. *thermocellum* were significantly higher than those of treatment without *C*. *thermocellum*. The *H* value of treatment with 1% *C*. *thermocellum* inoculum ratio was also the highest for methanogens in solid phase as in liquid phase, and showed a significant increase from Day 5 to Day 20. The *H* values of treatment with 0% bacterial inoculum ratio were almost stable for the duration. However, the *H* values of treatment with 5% and 10% inoculum ratios were close to those of treatment with 0% inoculum ratio during the first 12 days and then exhibited an obvious decrease.

## Discussion

The cells of *Chlorella* are surrounded by a recalcitrant cellulosic cell wall, which encloses a parietal and cup-shaped chloroplast with a pyrenoid [9,22]. While stained with multiple fluorochromes, the *C*. *vulgaris* cell wall consisted of β-polysaccharides could be clearly observed (Additional file 1: Figure S5 and Figure S6) [Note: cellulose belongs to β-polysaccharides]. If the microalgal biomass is not subjected to any cell disruption process, then the cell walls could be very resistant to hydrolysis, protecting the cells against the enzymes produced by the anaerobic consortium, and thus restricting cell biodegradability. *C*. *thermocellum* is an acetogenic, thermophilic, and anaerobic bacterium with a high rate of cellulose degradation and propensity to synthesize hydrogen. The present study found that *C*. *thermocellum* could utilize *C*. *vulgaris* as a substrate for growth (Figure 4). Furthermore, addition of *C*. *thermocellum* resulted in the production of higher levels of hydrogen (Figure 1), along with higher concentrations of ethanol, acetate, and butyrate (Figure 2). These results imply the contribution of *C*. *thermocellum* to algal cell degradation (Figure 5). The improved cell wall breakage resulted in the release of more organic matter, thus enhancing the diversity of bacteria in both suspension and granular phases, and the diversity of methanogens in the suspension phase (Figure 6). In addition, their quantities (as suggested by more SMP in Figure 4) were also ameliorated, favoring improvement in fermentation efficiency and process stability. Meanwhile, the hydrogen generated from *C*. *thermocellum* activity could promote the development of hydrogenotrophic methanogenesis, resulting in higher methane yield (Figure 1, Table 1) and an increase in the abundance of hydrogenotrophic methanogens, thus reducing the diversity of methanogens in granular phase (Figure 6). Therefore, anaerobic digestion of *C*. *vulgaris* biomass could be improved by the addition of *C*. *thermocellum* through enhanced cell disruption and excess hydrogen production.

The methane yield achieved from *C*. *vulgaris* degradation without bioaugmentation was 322 ml CH_4_/g VS, which is equivalent to 50% of the theoretical methane yield estimated by the carbohydrates, proteins, and lipid content of *C*. *vulgaris* and calculated according to Becker [23]. CH_4_ yield from microalgae was bound up with chemical composition of microalgal biomass and process parameters such as the bioreactor type and the digestion temperature [16]. Lakaniemi et al. [11] reported 286 ml CH_4_/g VS from *C*. *vulgaris* at 37°C. Bruhn et al. [24] reported 271 ml CH_4_/g VS from macerated *Ulva lactuca* at 52°C and found that a decrease of the digestion temperature from 52°C to 37°C lowered the final methane yield by 7%. Thus, the CH_4_ yield from *C*. *vulgaris* without bioaugmentation was comparable with previous results. With the addition of *C*. *thermocellum*, the methane production could be further increased by 17?~?24%.

Unlike the one-step process, hydrogen accumulated in the cultures inoculated only with *C*. *thermocellum* in the first stage of the two-step treatment was not consumed by methanogens. As a result, both the bioenergy gases, hydrogen and methane, could be recovered. In Series 2, the hydrogen and methane yield from two-step treatment was 53 and 321 ml/g VS, respectively, equivalent to 13.4 kJ/g VS of energy, which is 9.4% higher than the corresponding yield obtained from one-step treatment. When compared with the hydrogen yield reported in the literature, the yield obtained in the present study is of average magnitude. For example, Park et al. [25] reported a hydrogen yield of 28 ml H_2_/g dry weight from the microalgae *Laminaria japonica* pretreated by ball milling and heat treatment at 120°C for 30 min, using anaerobic sewage sludge as an inoculum. Yang et al. [26] achieved a hydrogen yield of 27.27 ml H_2_/g VS from lipid-extracted *Scenedesmus* biomass subjected to heat pretreatment at 95°C for 30 min. Carver et al. [27] reported hydrogen yields of 82 and 114 ml H_2_/g VS from *C*. *vulgaris* using microalgae-associated bacteria and a thermophilic consortium at 60°C, respectively, while Lakaniemi et al. [11] obtained a much lower hydrogen yield of 10.8 ml H_2_/g VS from the same algal biomass at 37°C.

It should be noted that bioaugmentation with *C*. *thermocellum* made the anaerobic digestion system complex. The activity of this acidogenic phase bacteria might be coupled with a probable inhibition of the methanogens and/or a slower rate of acid intermediate consumption by the methanogenic process [4]. The longer lag time of *C*. *thermocellum* at a high inoculum ratio could probably be due to the need for the methanogens to alter their physiological state according to the new environment [28]. In addition to higher production of hydrogen, the two-step treatment presented shortest lag time and a comparable level of methane production, providing an energy efficiency method worthy of consideration. The proportions of algal biomass and methanogenic inoculum may also be an important parameter for methane production. The low proportion of granular sludge resulted in a much lower methane yield, longer lag time, and lower maximum methane production rate. This might be due to low methanogenic activity or the number of methanogens, which could result in the accumulation of VFAs. Therefore, proper methanogenic inoculum ratio relative to the amount of algal biomass and *C*. *thermocellum* should be considered.

## Conclusions

Bioaugmentation with *C*. *thermocellum* improved the degradation of *C*. *vulgaris* biomass, producing higher levels of methane and hydrogen. However, the increases in methane yield were in the same order of magnitude with different inoculum ratios of *C*. *thermocellum*. The two-step process, with methanogenic inoculum added after the hydrogen production reached saturation, was found to be an energy-efficiency method for hydrogen and methane production. The fluorescence peaks of EEM spectra associated with chlorophyll can serve as biomarkers for algal cell degradation.

## Methods

### Substrate and inoculum

The microalgae *C*. *vulgaris* (strain ESP-6, Department of Chemical Engineering, National Cheng Kung University, Tainan, Taiwan) were grown photoautotrophically in Liquid Bold’s Basal Medium (BBM) [29] with 0.1 vvm CO_2_ (5% CO_2_ and 95% 0.45-μm filtered air) sparging. Light was provided by 8000–10000 lux LED lights (WD-TM-D35W, Widen Photodiode Technology Co., China). After 7 days of incubation, the microalgal biomass was harvested and concentrated by centrifugation at 3600?×?*g* for 15 min. The solid concentrate was subjected to anaerobic digestion. The concentrated algal biomass contained 12.9% (on wet weight basis) of total solid (TS), 93.5% (on dry weight basis) of volatile solid (VS), and 58, 11, and 14% (on dry weight basis) of proteins, lipids, and sugars, respectively.

*C*. *thermocellum* (strain DSM2360) was obtained from Leibniz Institute DSMZ-German Collection of Microorganisms and Cell Cultures. Fresh cultures were maintained by routinely transferring 5% (v/v) inoculum into fresh medium containing 5 g/L of absorbent cotton. Other compounds contained in the fresh medium included (per liter of distilled water): KH_2_PO_4_, 0.50 g; K_2_HPO_4_?·?3H_2_O, 1.00 g; urea, 2.00 g; MgCl_2_?·?6H_2_O, 0.50 g; CaCl_2_?·?2H_2_O, 0.05 g; FeSO_4_?·?7H_2_O, 1.25 mg; morpholinopropane sulfonic acid, 10.00 g; resazurin, 1.00 mg; yeast extract, 6.00 g; glucose, 5.00 g; cysteine-HCl?·?H_2_O, 1.00 g. *C*. *thermocellum* was freshly harvested after 4 days of incubation when no more hydrogen production was detected.

Granular sludge, cultivated in a laboratory-scale (3.5 L) anaerobic sequenced batch reactor (ASBR), was added as the methanogenic inoculum. The ASBR was operated at 55°C, and glucose and acetate (80%:20%, calculated as COD) were utilized as the feedstock at an organic loading rate of 2 g COD/(L-day). The methanogenic sludge was taken after being acclimated for more than 50 days and rinsed with anaerobic preheated (55°C) buffer solution to remove the residual carbon. The buffer solution was the same as that used in the subsequent batch experiments. The TS of the granular sludge was 11.1% (w/w) and the VS was 77.8% (w/w) of the TS.

### Experimental setup

(1) Series 1: One-step methane production with different inoculum ratios of *C*. *thermocellum*

Batch experiments were conducted at 55°C with 2 g VS/L of the algal biomass and 3 g VS/L of methanogenic sludge. The culture medium containing *C*. *thermocellum* was added at different inoculum ratios: 0%, 1%, 5%, and 10% (v/v). A control with only granular sludge and without microalgae was prepared to measure the endogenous activity of the sludge itself. Another control with granular sludge and culture medium containing 5% (v/v) of *C*. *thermocellum* was set up to determine the methane production potential of the culture medium. The bottles were filled up to 500 ml with buffer solution and flushed with nitrogen for 2 min to maintain anaerobic conditions. The composition of the buffer solution was as follows (per L): 1.0 g of NH_4_Cl, 0.4 g of K_2_HPO_4_?·?3H_2_O, 0.2 g of MgCl_2_?·?6H_2_O, 0.08 g of CaCl_2_?·?2H_2_O, 10 ml of trace element solution, and 10 ml of stock vitamin solution. The stock trace element and vitamin solutions were prepared according to Chen et al. [30].

(2) Series 2: Two-step co-production of hydrogen and methane

In the two-step experiment, 3 g VS/L of algal biomass was first incubated with 5% (v/v) of *C*. *thermocellum* containing culture medium at 55°C for 7 days in 500-ml buffer solution, as mentioned earlier, for hydrogen production. Subsequently, 2 g VS/L of methanogenic sludge was added to produce methane. As a reference, a one-step experiment with the same amount of algal biomass, granular sludge, and 5% (v/v) of *C*. *thermocellum* containing culture medium was set up. Furthermore, a control with granular sludge and 5% (v/v) of *C*. *thermocellum* containing culture medium, similar to that used in Series 1, was also included in this series. Two different inoculum to substrate ratios (methanogenic sludge to microalgal biomass: 3:2 in series 1 and 2:3 in series 2) were introduced to investigate the influence of inoculum to substrate ratio. All the experiments were carried out in duplicate and the results were expressed as means.

### Analysis of gaseous and liquid samples

Gas production was measured by manometric methods. The pressure in the headspace of the serum bottles was measured by a Testo 512 pressure meter (Testo, Germany). The concentrations of hydrogen, methane_,_ and carbon dioxide in the biogas were analyzed using a gas chromatograph (GC112A, Shanghai Precision & Scientific Instrument Co., China) equipped with a thermal conductivity detector (TCD). The gas volumes were corrected to standard temperature and pressure conditions (STP: 0°C and 1013 kPa). Methane production from the culture medium and methanogenic sludge was deducted in the reported data. Gompertz modeling (Eq.1) was used according to Lü et al. [31] to fit the curve of the cumulative methane production, and the values of three parameters (*P*, *R*_*max*,_ and λ) were determined.

(1) $$M\left(t\right)=P∙\exp\{-\exp\left[\frac{R_{\max}∙e}{P}\left(\lambda -t\right)+1\right]\;\}$$

*M*(*t*) is the cumulative methane production (ml/g VS added) at time *t* (days), *P* is the highest methane yield (ml/g VS), *R*_max_ is the maximum methane production rate (ml/g VS/day), and *λ* is the lag phase (days). Lag phase refers to the initial adaptive phase, during which methane production remains relatively constant prior to rapid growth.

The liquid samples were centrifuged at 16,000?×?*g* for 10 min. Subsequently, the supernatants were collected and analyzed for pH, volatile fatty acids (VFAs), alcohols, dissolved organic carbon (DOC), total inorganic carbon (TIC), dissolved nitrogen (DN), and three-dimensional fluorescent intensity. The pH was tested with a pHS-2 F Digital Meter. The DOC, TIC, and DN were analyzed on a TOC-V_CPH_ Analyzer (Shimadzu, Japan). The concentrations of VFAs (including acetic, propionic, isobutyric, butyric, and isovaleric acids) and alcohols in the supernatant were determined using an Agilent 6890 N gas chromatography (GC) system equipped with a flame ionization detector (FID). The fluorescence excitation-emission matrixes (EEM) were recorded for the supernatant in a 10-mm quartz cuvette in a Varian Cary Eclipse fluorometer (Agilent, Santa Clara, CA, USA). The emission was scanned from 220 to 750 nm at 2-nm intervals and 10-nm bandwidth, while the excitation was produced with a Xenon flash lamp in 10-nm bandwidth at 10-nm intervals from 200 to 700 nm. The EEM signals were processed and subjected to parallel factor analysis (PARAFAC), as described in the study by Lu et al. [32].

### Transmission electron microscopy observation of the microalgal cell

The cells of *C*. *vulgaris* were observed using transmission electron microscopy (TEM). Samples were prepared according to the procedure developed by Yamamoto et al. [33], and examined with a transmission electron microscope (JEM-1230, JEOL, Japan).

### Multiple fluorochrome staining of the microalgal cell and spectral microscopy observation

The cells of *C*. *vulgaris* were stained successively by FITC for proteins, Con A for α-polysaccharides and calcofour white for β-polysaccharides according to Chen et al. [34]. The samples were then examined with a Leica DMI 4000B spectral microscope imaging system.

### DNA manipulation

Both the liquid samples and granules corresponding to different sampling dates were used for DNA extraction. The total DNA was extracted from the pellets using PowerSoil DNA isolation kit (MoBio Laboratories Inc., CA), according to the manufacturer’s protocol. The fingerprint technique of Automated Ribosomal Intergenic Spacer Analysis (ARISA) was used to monitor the microbial dynamics. The extracted DNA was amplified using primers 1389 F and 71R for archaea, and primers ITSF and ITSReub for bacteria, respectively. Polymerase chain reaction (PCR) and ARISA of the PCR product were carried out according to the method described by Qu et al. [35]. Shannon diversity index was used to analyze the ARISA profiles and *H* value was calculated using the software PAlaeontological STatistics (PAST) version 2.17b, according to the procedure proposed by Hammer et al. [36].

## Abbreviations

VFA: Volatile fatty acid; EEM: Excitation-emission matrix; PARAFAC: Parallel factor analysis; SMP: Soluble microbial products; TEM: Transmission electron microscopy; ARISA: Automated ribosomal intergenic spacer analysis; PCR: Polymerase chain reaction; DOC: Dissolved organic carbon; TIC: Total inorganic carbon; DN: Dissolved nitrogen; TS: Total solid; VS: Volatile solid.

## Competing interests

The authors declare that they have no competing interests.

## Authors’ contributions

FL conceived the study, participated in microbial community analyses, parallel factor analyses, data interpretation and thoroughly reviewed the manuscript. JJ carried out the microalgae and biomass production and harvesting, anaerobic cultivations and all related analyses, microbial community analyses, data interpretation, and drafting and completion of the manuscript. LS participated in the design of the study and data interpretation, and thoroughly reviewed the manuscript. PH participated in the design of the study and data interpretation, and thoroughly reviewed the manuscript. All authors read and approved the final manuscript.

## Supplementary Material

Additional file 1 Figure S1 ARISA profile of bacteria in liquid phase. The marks (e.g. “23”) above represent the time of incubation, for example, “23” means the day 23 in the incubation. **Figure S2** ARISA profile of bacteria in solid phase. The marks (e.g. “12”) above represent the time of incubation, for example, “12” means the day 12 in the incubation. The profile marked with 0 represents the profile of the seed sludge, which was used in all the reactors at day 0. **Figure S3** ARISA profile of methanogens in liquid phase. The marks (e.g. “23”) above represent the time of incubation, for example, “23” means the day 23 in the incubation. **Figure S4** ARISA profile of methanogens in solid phase. The marks (e.g. “12”) above represent the time of incubation, for example, “12” means the day 12 in the incubation. The profile marked with 0 represents the profile of the seed sludge, which was used in all the reactors at day 0. **Figure S5** The spectral microscope images of stained *Chlorella vulgaris*. **a** phase contrast photograph, **b** combined image of individual images in c-e, **c** spectral microscope image of α-polysaccharides (con A), **d** spectral microscope image of β-polysaccharides (calcofluor white), **e** spectral microscope image of protein (FITC). **Figure S6** The spectral microscope images of stained *Chlorella vulgaris* cells and cell walls. **a** phase contrast photograph, **b** spectral microscope image of β-polysaccharides (calcofluor white). Click here for file
