# Supplementary material for: Bacterial bioaugmentation for improving methane and hydrogen production from microalgae
Source: Biotechnol Biofuels. 2013 Jul 1;6:92. doi: 10.1186/1754-6834-6-92 (PMC3699423; doi:10.1186/1754-6834-6-92)
Supplement: Additional file 1 Figure S1 — ARISA profile of bacteria in liquid phase. The marks (e.g. “23”) above represent the time of incubation, for example, “23” means the day 23 in the incubation. Figure S2 ARISA profile of bacteria in solid phase. The marks (e.g. “12”) above represent the time of incubation, for example, “12” means the day 12 in the incubation. The profile marked with 0 represents the profile of the seed sludge, which was used in all the reactors at day 0. Figure S3 ARISA profile of methanogens in liquid phase. The marks (e.g. “23”) above represent the time of incubation, for example, “23” means the day 23 in the incubation. Figure S4 ARISA profile of methanogens in solid phase. The marks (e.g. “12”) above represent the time of incubation, for example, “12” means the day 12 in the incubation. The profile marked with 0 represents the profile of the seed sludge, which was used in all the reactors at day 0. Figure S5 The spectral microscope images of stained Chlorella vulgaris. a phase contrast photograph, b combined image of individual images in c-e, c spectral microscope image of α-polysaccharides (con A), d spectral microscope image of β-polysaccharides (calcofluor white), e spectral microscope image of protein (FITC). Figure S6 The spectral microscope images of stained Chlorella vulgaris cells and cell walls. a phase contrast photograph, b spectral microscope image of β-polysaccharides (calcofluor white). [file 1754-6834-6-92-S1.docx]

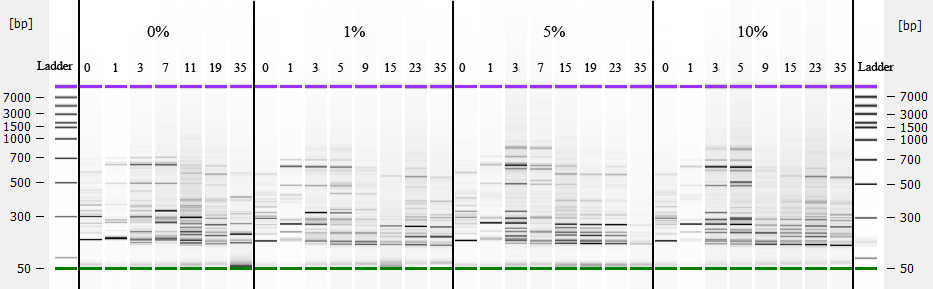


**Figure S1. ARISA profile of bacteria in liquid phase.** The marks (e.g. 23) above represent the time of incubation, for example, 23 means the day 23 in the incubation.


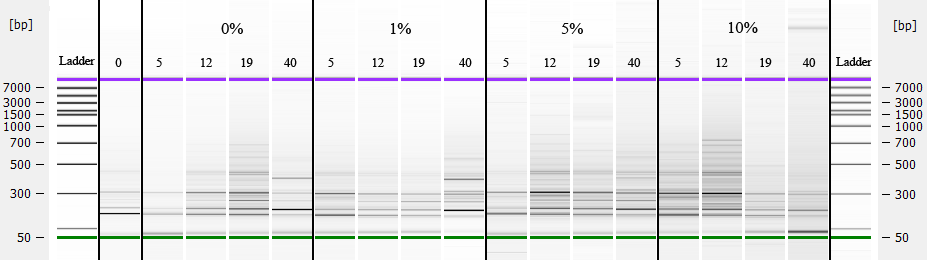


**Figure S2. ARISA profile of bacteria in solid phase.** The marks (e.g. 12) above represent the time of incubation, for example, 12 means the day 12 in the incubation. The profile marked with 0 represents the profile of the seed sludge, which was used in all the reactors at day 0.


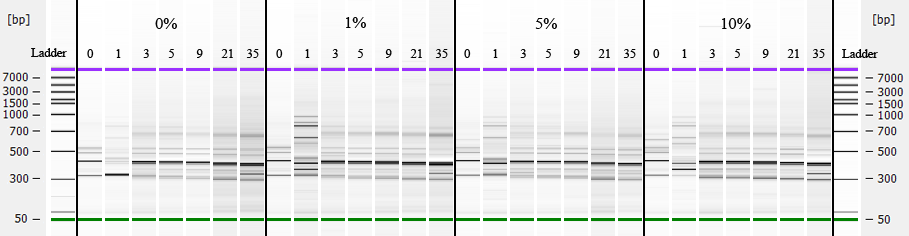


**Figure S3. ARISA profile of methanogens in liquid phase.** The marks (e.g. 23) above represent the time of incubation, for example, 23 means the day 23 in the incubation.

**
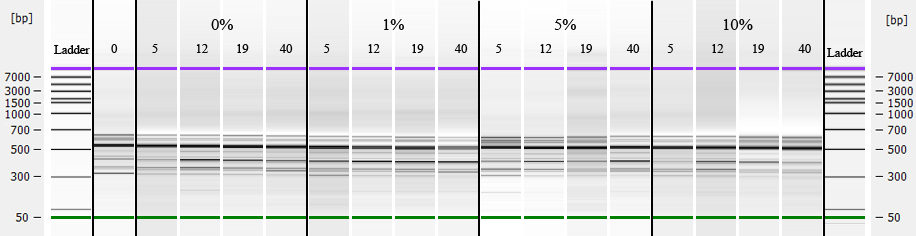
**

**Figure S4. ARISA profile of methanogens in solid phase.** The marks (e.g. 12) above represent the time of incubation, for example, 12 means the day 12 in the incubation. The profile marked with 0 represents the profile of the seed sludge, which was used in all the reactors at day 0.


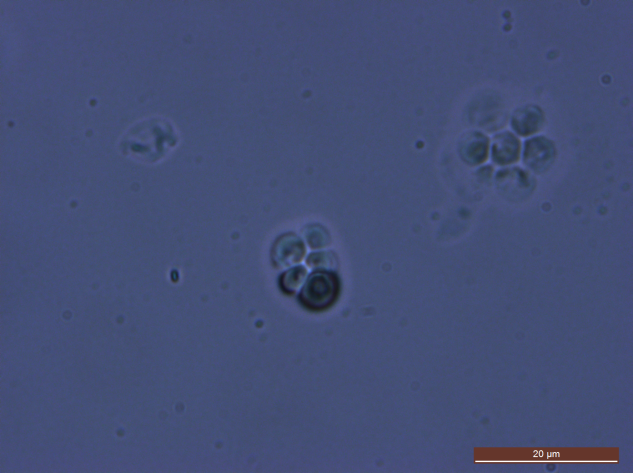

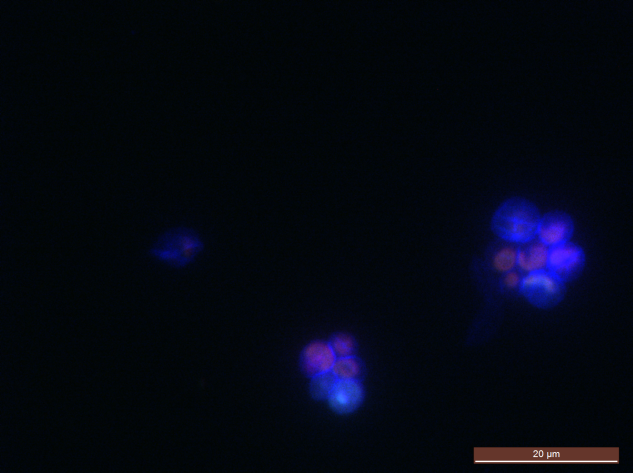

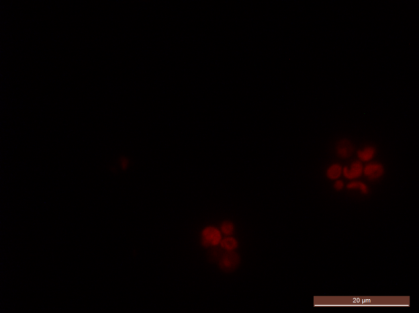

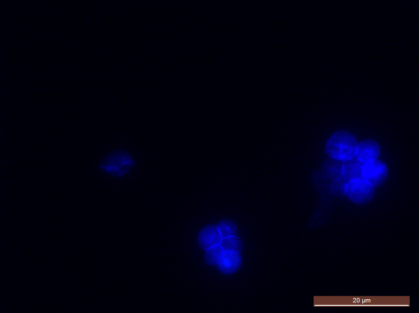

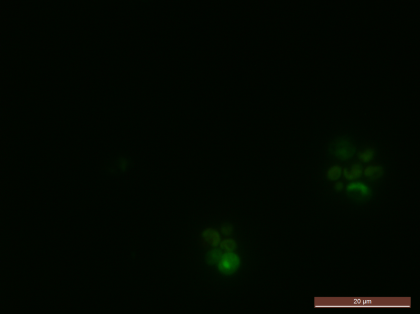


**e**

**d**

**c**

**b**

**a**

**Figure S5. The spectral microscope images of stained** ***Chlorella vulgaris*.** **a** phase contrast photograph, **b** combined image of individual images in c-e, **c** spectral microscope image of α-polysaccharides (con A), **d** spectral microscope image of β-polysaccharides (calcofluor white), **e** spectral microscope image of protein (FITC).


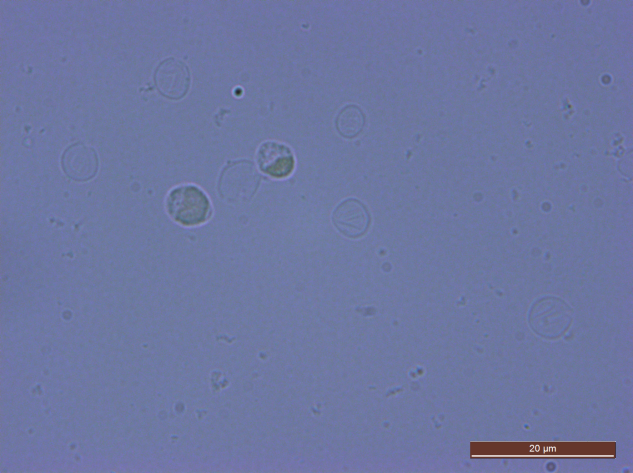

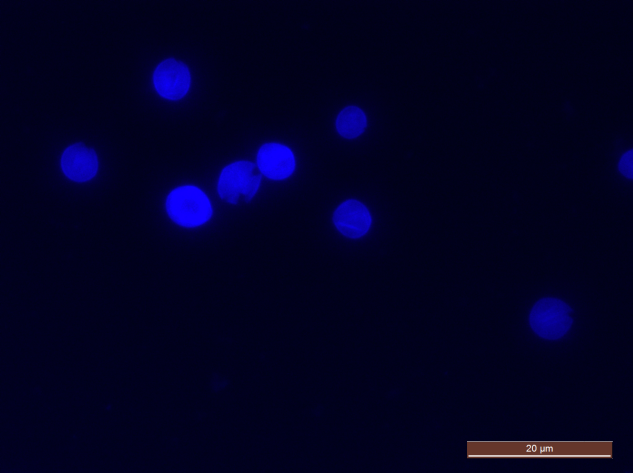


**b**

**a**

**Figure S6. The spectral microscope images of stained *Chlorella vulgaris* cells and cell walls.** **a** phase contrast photograph, **b** spectral microscope image of β-polysaccharides (calcofluor white).
